# Contribution of Liver and Pancreatic Islet Crosstalk to β-Cell Function/Dysfunction in the Presence of Fatty Liver

**DOI:** 10.3389/fendo.2022.892672

**Published:** 2022-05-16

**Authors:** Lucía López-Bermudo, Amparo Luque-Sierra, Douglas Maya-Miles, Rocío Gallego-Durán, Javier Ampuero, Manuel Romero-Gómez, Genoveva Berná, Franz Martín

**Affiliations:** ^1^ Andalusian Center of Molecular Biology and Regenerative Medicine (CABIMER), University Pablo Olavide, University of Seville, CSIC, Seville, Spain; ^2^ Biomedical Research Network on Diabetes and Related Metabolic Diseases (CIBERDEM), Instituto de Salud Carlos III, Madrid, Spain; ^3^ Hospital Universitario Virgen del Rocío de Sevilla, Instituto de Biomedicina de Sevilla, Universidad de Sevilla, Sevilla, Spain; ^4^ Biomedical Research Network on Hepatic and Digestive Diseases (CIBEREHD), Instituto de Salud Carlos III, Madrid, Spain

**Keywords:** β-cell, pancreatic islets, liver, hepatokines, crosstalk, type 2 diabetes, nonalcoholic fatty liver disease, extracellular vesicles (EVs)

## Abstract

Tissue-to-tissue crosstalk regulates organ function, according to growing data. This phenomenon is relevant for pancreatic β-cells and the liver, as both tissues are involved in glucose homeostasis and lipid metabolism. The ability to fine-tune regulation and adaptive responses is enabled through communication between pancreatic β-cells and the liver. However, the crosstalk between both tissues changes when metabolic dysregulation is present. Factors and cargo from extracellular vesicles (EVs) released by liver and pancreatic β-cells that reach the circulation form the words of this interaction. The molecules released by the liver are called hepatokines and are usually secreted in response to the metabolic state. When hepatokines reach the pancreatic islets several mechanisms are initiated for their protection or damage. In the case of the crosstalk between pancreatic β-cells and the liver, only one factor has been found to date. This protein, pancreatic derived factor (PANDER) has been proposed as a novel linker between insulin resistance (IR) and type 2 diabetes mellitus (T2D) and could be considered a biomarker for non-alcoholic fatty liver disease (NAFLD) and T2D. Furthermore, the cargo released by EVs, mainly miRNAs, plays a significant role in this crosstalk. A better knowledge of the crosstalk between liver and pancreatic β-cells is essential to understand both diseases and it could lead to better prevention and new therapeutic options.

## Introduction

To coordinate their metabolic response to changes in their environment, organisms need to establish communication systems between their constituent tissues. These dialogs between organs allow fine-tuned regulation and adaptive response. Currently, it is known that an alteration in these communication channels contributes to the appearance of multiple pathologies. Among the tissues that communicate with each other, the most common are those responsible for the maintenance of energetic and metabolic homeostasis. This conversation brings together multiple organs (liver, muscle, adipose tissue, pancreas, digestive system and brain) to maximize the efficiency of the processes responsible for maintaining homeostasis and energy balance. In this type of communication, the words are formed by the factors secreted by the tissues that reach the bloodstream and from there goes to various organs to foster the communication. These factors can range from small molecules to peptides and even hormones. Presumably, these words will vary depending on the pathophysiological situation of the tissues sending the messages. Finding the meaning of these words is important in elucidating the pathophysiology of metabolic diseases. In the case of communication between the liver and the pancreas, the messages sent by the liver are called hepatokines. In contrast, the words used by the pancreas have not yet been given a specific name.

The crosstalk between the liver and pancreas plays an essential role in glucose and lipid metabolism and is therefore central to the ability of organisms to respond to changing nutritional states. Given the critical functions of the liver, declining metabolic health is unsurprisingly one of the first indications of metabolic disease. Hepatic steatosis is correlated with T2D and is an early indicator of IR (1).

T2D and NAFLD often occur together, as both share similar pathogenic mechanisms. In fact, NAFLD has a prevalence of approximately 70-80% in T2D patients (2). The relationship between T2D and NAFLD was first described a decade ago in Pima Indians with T2D (3). T2D has been shown to be an independent predictor factor for the progression of NAFLD to non-alcoholic steatohepatitis (NASH) and liver fibrosis (4). However, NAFLD is also associated with a higher risk of incident T2D and diabetes-related complications (5). The relationship between both metabolic diseases could be considered a vicious cycle because NAFLD is associated with a higher risk of developing T2D, and patients with T2D have a higher prevalence of NAFLD.

The link between NAFLD and IR has been extensively described and characterized (6). When T2D is diagnosed, patients have hyperglycemia, IR and β-cell function impairment. Both pathologies are closely related, and before overt T2D appears, the pancreas responds to increased IR by releasing more insulin, while the liver decreases insulin clearance to boost plasmatic insulin concentrations and prevent T2D onset. However, frequently, the higher insulin concentrations are insufficient due to β-cell dysfunction, which is caused by an increased β-cell workload and the effects of glucolipotoxicity. Finally, when β-cells are unable to overcome peripheral IR, patients develop T2D. However, whether NAFLD is the cause, the consequence, or both of IR is still unknown.

In this review, we will describe the crosstalk between liver cells and insulin producing β-cell and how liver seems to detect the loss of β-cell function and cell mass and release molecules to communicate with pancreatic β-cell. This communication can contribute to improve or impairs islet function. On the other way round, we will illustrate, that in situations of IR, pancreatic β-cells increase the release of a cytokine that binds the liver promoting gluconeogenesis and steatosis.

## Hepatokines and Factors Secreted by the Liver That Mediate Communication With Pancreatic Islets

Hepatokines are proteins secreted by hepatocytes responsible for regulating energy homeostasis and may affect metabolism in distant tissues. These proteins are usually secreted in response to the metabolic state. Many hepatokines have been linked to the induction of metabolic dysfunction (7). The fatty liver secretome has been suggested to contribute to the appearance of a diabetogenic milieu throughout the release of hepatokines (8). In this sense, evidence is accumulating showing that an increase in the presence of lipids in the liver initiates the secretion processes of numerous hepatokines (8).

This process begins with increased energy availability, which leads to lipid accumulation in the liver and local infiltration and activation of immune cells (9). As a consequence, the liver releases proinflammatory factors that reach the tissues and exert their effects. Some of these hepatokines, which regulate the metabolism of the organism, reach the pancreatic islets and initiate a series of mechanisms for their protection or damage.

Here, we will focus on the hepatokines and factors released by the steatotic liver that have been found to regulate pancreatic islet cell function and mass as a consequence of organ crosstalk (**Table 1**).

**Table 1:** Hepatokines released by fatty liver and their role in liver and pancreatic islet crosstalk.

Hepatokine	Expression in liver metabolic disease	Effect on pancreatic islet
ANGPTL8	Increased	Improves β-cell proliferation
		Improves GSIS
Fetuin A	Increased	Decreases β-cell proliferation
		Decreases GSIS
FGF21	Increased	Improves β-cell proliferation
		Improves GSIS
		Decreases β-cell apoptosis
Follistatin	Not modified	Decreases β-cell apoptosis
		Improves β-cell proliferation
HGF	Increased	Improves β-cell proliferation
		Improves GSIS
IGFBPs	Decreased	Improves β-cell proliferation
KISS1	Increased	Improves GSIS
Serpin B1	Decreased	Improves β-cell proliferation
SHBG	Decreased	Not clear action
SeP	Increased	Increases β-cell apoptosis
		Decreases GSIS

### Angiopoietin Like 8

Angiopoietin like 8 (ANGPTL8) is also known as refeeding induced fat and liver, lipasin or betatrophin. This hormone regulates serum triglyceride levels and is probably involved in the transition between fasting and refeeding (10). Recently, several human studies have shown that serum ANGPTL8 levels are increased in patients with obesity (11), T2D (11) and NAFLD (12). Concerning its role in β-cell physiology, ANGPTL8 has been shown to induce pancreatic β-cell proliferation and insulin release in an insulin-deficient mouse model of IR (13).

### Fetuin-A

This protein is part of the fetuin family. Fetuins are involved in the transport of several substances in the bloodstream. Fetuin-A mainly binds free fatty acids (14). This protein is secreted by the liver, and its release is higher in fatty liver hepatocytes (15). It has been shown that fetuin-A increases adipose tissue inflammation *via* activation of Toll-like receptor 4 (TLR4) (14). Thus, this protein is partly responsible for IR and T2D.

Gerts et al. showed that fetuin-A inhibited glucose-stimulated insulin secretion (GSIS) in adult human islets (16). In another study, Gerts et al. identified its mechanism of action (17). Fetuin-A retards pancreatic islet functional maturation, impairing the TGFBR-SMAD2/3 signaling pathway (17). Moreover, the protein downregulates the expression of FOXM1 and its target genes, restraining β-cell adaptive proliferation. It has been shown that fetuin-A decreases peripartum. This decrease contributes to postnatal maturation and proliferation of neonatal pancreatic islet cells. During adult life, the presence of liver steatosis generates increases in fetuin-A release, which impairs the function of β-cells and the adaptive increase in their mass in response to IR. This last event precipitates the onset of T2D.

### Fibroblast Growth Factor 21

Fibroblast growth factor (FGF) 21 is an endocrine factor that belongs to the FGF family. It is a regulator of glycemia, lipid metabolism and energy homeostasis. Studies in animal models of metabolic diseases have shown that FGF21 can reduce plasma glucose and insulin levels and triglycerides, as well as improve insulin sensitivity and glucose clearance. Moreover, in the presence of a high-fat diet (HFD), FGF21 prevented weight gain and liver steatosis in mice (18, 19). Patients with NAFLD (20), steatohepatitis (NASH) (21), obesity (21) and T2D (21) displayed much higher FGF21 serum concentrations. In several clinical trials, FGF21 analogs have been shown to improve liver health (22). This result suggests that in the future, FGF21 analogs could be used as therapeutic agents for NASH.

Pancreatic islets are an important target for FGF21. In an animal model of diabetes, FGF21 improved β-cell function and survival (23). Moreover, in FGF21-KO mice, altered islet morphology and impaired GSIS were observed. These effects are probably meditated *via* modulation of growth hormone (GH) signaling (24). Finally, in db/db mice, downregulation of FGF21 expression was observed (25). This finding could indicate a role for FGF21 in maintaining insulin homeostasis and islet β‐cell function. In db/db mice, FGF21 knockout increased lipid-induced islet β‐cell failure and suppressed GSIS. In contrast, pancreatic FGF21 overexpression significantly increased insulin expression, enhanced GSIS, improved islet morphology and reduced β‐cell apoptosis. The FGF21 mechanism of action indicates an increase in insulin gene transcription factors and soluble N‐ethylmaleimide‐sensitive factor attachment protein receptor (SNARE) proteins, as well as the activation of islet phosphatidylinositol 3‐kinase (PI3K)/Akt signaling (25). Other signaling pathways involved are i) the upregulation of carnitine palmitoyltransferase 1 (*CPT1*) expression and the downregulation of sterol regulatory element binding transcription factor 1 (*SREBF1*) and fatty acid synthase (*FASN*) expression, which decreases lipid accumulation in β-cells; ii) the restoration of the expression of genes responsible for the differentiation and maintenance of the β-cell phenotype [pancreatic and duodenal homeobox 1 (*PDX1*), insulin (*INS*) and MAF BZIP transcription factor A (*MafA*)] and iii) the reduction of immune cell infiltration and activation within islets (22).

### Follistatin

Follistatin is a monomeric glycosylated protein, expressed in several tissues, including the liver, that binds and neutralizes activins with high affinity and neutralizes their bioactivity. Follistatin also binds with lower affinity to other members of the transforming growth factor-β superfamily including myostatin and bone morphogenic proteins 2, 5, 7, and 8 (26). Activin A plays an important role in pancreatic development and homeostasis (27). Its production is increased in many acute and chronic inflammatory conditions stimulated by inflammatory cytokines, toll-like receptor ligands, and oxidative stress (28). Activin A can decrease the number of mature β-cells and increase the number of immature β-cells when essayed on primary mouse islets.

Follistatin has been linked to T2D and NAFLD. In this regard, higher levels of follistatin have also been recently associated to a higher risk of developing T2D, independently of established risk factors (29). Hansen et al. have shown that the liver is a major contributor to circulating follistatin in response to exercise. This increase in its expression by hepatocytes seems to be regulated by glucagon and insulin, which can either promote or inhibit its expression both, *in vivo* and *in vitro.* Authors also show that short-term follistatin treatment reduces glucagon secretion from pancreatic islets, whereas long-term follistatin treatment prevents apoptosis and induces proliferation of rat β-cells (30). Overexpression of follistatin in the pancreas of db/db mouse increases β-cell islets mass and decreases fasting glucose levels. Islet enlargement in this context seems to be attributed to β-cell proliferation resulting from the bioneutralization of myostatin and activin A by this hormone (31). Follistatin gene delivery has also recently shown to be able to promote insulinemia and abundance of insulin-positive pancreatic β-cells in mice, even when the treatment is administered to mice with advanced diabetes, supporting a mechanism for improved glycemic control associated with the maintenance of functional β-cells (32).

### Hepatic Growth Factor

Hepatic growth factor (HGF) is a mesenchyme-derived factor that acts as a circulating molecule involved in liver regeneration after hepatic damage. More recently, HGF was shown to be a pleiotropic factor that promotes cell survival and tissue regeneration and improves chronic inflammation and fibrosis in a wide variety of tissues (33).

Serum HGF levels are associated with liver diseases, obesity and IR. Almost two decades ago, Balaban et al. showed that NASH patients had higher serum HGF levels, although the observed increase was not significant (34). In a mouse model of HFD-NASH, Li et al. showed that HGF ameliorated liver steatosis and inflammation by activating the Janus kinase (JAK) 2- signal transducer and activator of transcription (STAT) 3 signaling pathway (35).

In the case of pancreatic islets, HGF has been observed in islets of different species (36). Moreover, García-Ocaña et al. showed that HGF is a mitogen and an insulinotropic agent for fetal and adult islet cells *in vitro* (36). To do this, the authors developed transgenic mice overexpressing HGF in the islet under rat insulin promoter (RIP) control. These researchers observed that the RIP-HGF mice had increased islet sizes and β-cell numbers. Moreover, the mice displayed lower fasting and non-fasting blood glucose concentrations, higher plasma insulin levels and better glucose tolerance than their normal littermates. The pancreatic islets of these mice showed better GSIS (36). In addition, HGF could increase the expression of regenerating protein, a protein implicated in pancreatic regeneration, in human fetal islets (37). Another study demonstrated that intraperitoneal administration of HGF in diabetic mice improved blood glucose levels after transplantation of an insufficient number of pancreatic islets (38). These studies suggest that HGF can improve the quantity, function and survival of pancreatic islets. Other studies using a HGF receptor KO mouse model have shown that when HGF is inhibited in pancreatic β-cells. In HGF receptor KO animals, there was a reduction in β-cell GLUT-2 expression, a reduction in insulin secretion and a decrease in glucose tolerance. However, total β-cell mass, proliferation and islet morphology did not change (39). In contrast, Dai et al. showed a reduction in the size of the islets, together with lower circulating insulin levels and moderate hyperglycemia (40). Álvarez-Perez et al. showed that HGF signaling is required for β-cell regeneration after β-cell ablation (41). In a model of T2D (insulin receptor substrate (IRS) KO mice), β-cell HFG overexpression compensated for the negative effects related to the absence of IRS2 by normalizing β-cell mass and improving glucose homeostasis (42). Finally, in obesity, pancreatic β-cells release more insulin to compensate for IR and in an attempt to normalize blood glucose levels. The observed hyperinsulinemia is the first step toward the onset of T2D. Because HGF improves GSIS and β-cell mass and because HGF plasma levels are higher in obesity, it has been suggested that HGF might link IR and β-cell hyperplasia (43).

### Insulin-Like Growth Factor Binding Proteins

Insulin-like growth factor binding proteins IGFBPs) are produced in several tissues, including the liver, in response to growth hormone (GH). The majority of circulating IGFBPs are synthesized in the liver (44). Recently, several IGFBPs have been implicated in the regulation of glucose homeostasis and NAFLD pathophysiology. In particular, IGFBP-7 could be used as a circulating marker of NAFLD severity. Finally, the same study suggested that IGFBP-3 and IGFBP-7 promote IR (45).

IGFBPs are crucial for insulin signaling. In this regard, low circulating IGFBP levels are associated with T2D. Moreover, in mice, an increase in IGFBP levels raises insulin sensitivity (46). Finally, in zebrafish, mouse and human pancreatic islets, IGFBP1 was shown to promote β-cell regeneration by inducing α-to-β-cell transdifferentiation (47). In fact, culturing mouse and human islets with recombinant IGFBP1 increased the number of cells co-expressing insulin and glucagon (47).

### Kisspeptin

Kisspeptins are a family of peptides encoded by the *KISS1* gene that have been identified as endogenous ligands for G-protein-coupled receptor 54 (GPR-54). *KISS1* was initially identified in breast cancer and melanoma cell lines and acts as a metastatic suppressor gene (48). In addition, this molecule plays an important role in the hypothalamus-pituitary gonadal axis regulating the onset of puberty (49). *KISS1* expression is increased in the liver and sera of patients with T2D and in mouse models of obesity and diabetes (50).

Hauge-Evans et al. demonstrated, in isolated mouse and human islets, that KISS1 potentiates GSIS in a glucose-dependent manner (51).

### Leukocyte-Neutrophil Elastase Inhibitor

Leukocyte-neutrophil elastase inhibitor, also known as serpin B1, has been identified as a hepatocyte-derived secretory protease inhibitor protein. A synthetic specific and competitive inhibitor of neutrophil elastase called sivelestat has shown protective effects in liver inflammatory states and ischemia and reperfusion injury (52). In addition, it has been shown that an increased ratio of neutrophil elastase and its inhibitor alfa1-antitrypsin is closely related to liver inflammation in patients with NASH (53).

In pancreatic islets, serpin B1 regulates mouse, zebrafish and human β-cell proliferation (54). In addition, sivelestat was shown to increase β-cell proliferation in cultured pancreatic islets and in transplanted islets (55). As serpin B1 is well conserved and has a defined activity among different species, it has potential as a therapeutic compound to promote β-cell regeneration.

### Sex Hormone-Binding Globulin

Sex hormone-binding globulin (SHBG) is a glycoprotein produced by the liver and responsible for the transport of sex steroid hormones (56). SHBG has been previously related to IR, liver lipid metabolism and NAFLD (57). Low serum levels of SHBG are associated with a higher prevalence of NAFLD in T2D patients (58). *In vitro*, it has been demonstrated that insulin inhibits hepatic SHBG synthesis (59). Moreover, several studies have shown a negative correlation between SHBG and insulinemia, indicating that SHBC could be considered a marker of IR, thereby predicting a later onset of T2D (60).

Reis et al. investigated the correlation between SHBG and pancreatic β-cell secretion in men with different body compositions. The researchers observed a negative correlation between SHBG circulating levels and pancreatic β-cell secretion (61). This study showed that in men, there exists a negative correlation between SHBG levels and β-cell function.

### Selenoprotein

Selenoprotein (SeP) is a selenium (Se)-rich plasma protein mainly produced in the liver. SeP functions as a Se-transport protein to deliver Se from the liver to other tissues (62). This protein play a pivotal role in Se metabolism and antioxidant defense (63). The majority of studies show that Se has a positive effect on liver steatosis, inflammation and fibrosis (64). In addition, SeP has been identified as a hepatokine that causes IR in T2D patients (65) and therefore was found to be increased in T2D patients (66). However, despite this, there are only a few studies reporting the association between SeP and liver diseases, such as NAFLD or NASH. In addition, these clinical studies show conflicting data among them. However, more studies have shown higher circulating levels in NAFLD and NASH patients (64). In fact, a recent study showed that in NAFLD patients, increased concentrations of SeP correlate with abdominal obesity and IR. Moreover, the study demonstrated that higher levels of SeP increase the risk of NAFLD development by 7.5 times (67).

In this regard, a decade ago, Steinbrenner et al. showed that SeP was present in α- and β-cells from mouse pancreatic islets. The islet-cell SeP expression was upregulated in the presence of high glucose concentrations and when islet-cells were subjected to oxidative stress conditions, such as streptozotocin. This suggest that SeP might act as an antioxidant to protect islet cells (68) from oxidative stress injury. However, more recent studies have shown that *in vivo* injection of SeP in T2D mouse models reduced GSIS, insulinemia and the area of pancreatic islets (69). There was also a decrease in the levels of β-cells and α-cells within pancreatic islets (69). All these data suggest that SeP affects pancreatic islet function and regeneration.

## Factors Secreted by Pancreatic Islets That Communicate With the Liver

The pancreatic islet can be considered a small organ, which is composed of a variety of endocrine cells and other cells of non-endocrine lineage that support the mini-organ. The functioning and activity of the pancreatic islet are determined by circulating glucose levels and by a complex variety of endocrine, paracrine, neuronal, and nutritional signals that act at multiple levels. Because the pancreatic islets are made up of various types of endocrine cells and their function and coordination between them are finely regulated, it is assumed that there is intense crosstalk within the pancreatic islets. Regarding intra-islet crosstalk, the relationship between α- and β-cells is the most studied aspect. More recently, δ cells have been shown to be important modulators of insulin and glucagon release (70).

In addition to communication within the islets, neighboring tissues, especially those that contribute to metabolic homeostasis, also participate in this dialog. In this sense, in the last decade, increasing evidence has shown that peripheral tissues such as adipose tissue, skeletal muscle, liver, intestine, bone, brain and the immune system send messages to the pancreatic islets (71).

Notably, all the communication that takes place within the interior of the pancreatic islet, as well as the conversation with other organs, changes under conditions of metabolic dysregulation. In fact, there is evidence indicating that many of the clinical manifestations that occur in metabolic diseases are due to a breakdown or modification of the communication already mentioned and that finely regulates the functioning of healthy pancreatic islets (72).

Notably, the dialog between the different tissues and the pancreatic islets is extensive and involves many partners. In contrast, the islet cells only appear to talk among themselves with little communication with the outside world. Although the islets are not very communicative, they receive a lot of information from the outside, but they do not communicate with the rest of the tissues of the organism. Therefore, it seems that communication is essentially unidirectional. The reasons for this finding may be threefold: i) this phenomenon has been little studied; ii) the islets do not have to communicate much; and iii) thus far we have not found many messengers from the pancreatic islets to the rest of the tissues. If we select the simplest explanation, which is usually the correct one, the most likely of all the reasons must be the third one.

In the case of communication between the pancreatic islets and the liver, a factor has been found called PANDER.

### Pancreatic Derived Factor

PANDER, also known as Family with Sequence Similarity 3 Member B (FAM3B), is a cytokine that has been implicated in multiple biological processes, among which the best-characterized is its role as a hormone in glucose and lipid metabolism (73). PANDER is a 235-amino acid protein with a secretion signal peptide (74). This protein is secreted together with insulin by pancreatic islet β-cell granules. Similar to insulin release, PANDER secretion also occurs in a Ca^2 +^ influx-dependent manner in pancreatic β cell lines and primary cultured mouse islets (75). PANDER secretion is also induced by glucose and is involved in glucose homeostasis (76, 77). Thus, PANDER could be involved in the regulation of insulin secretion. Recombinant PANDER treatment or PANDER gene overexpression induced pancreatic α and β cell apoptosis (77, 78). In addition, glucose, free fatty acids and pro-inflammatory cytokines were shown to induce PANDER gene expression in pancreatic β cells (79, 80). In pancreatic islets of db/db mice, PANDER mRNA and protein expression were increased. This expression was reversed by rosiglitazone and induced a decrease in hyperglycemia and IR (81). All of these findings indicate that PANDER has deleterious effects on pancreatic β cells under IR or hyperglycemic conditions. However, PANDER knockout mice exhibited impaired GSIS. Isolated islets from these animals also showed blunted insulin secretion when challenged with glucose (82). These observations suggest that PANDER has dual roles in pancreatic β cell functions. Under physiological conditions, PANDER improves insulin secretion, whereas in conditions of IR, an increase in PANDER expression and secretion exerts deleterious effects on pancreatic β cell functions.

Circulating PANDER was increased in patients with metabolic syndrome and could predict the risk of T2D in a Chinese population (83). It has been found that serum PANDER levels are negatively correlated with β-cell function in diabetic patients (84), indicating that PANDER is associated with β-cell dysfunction in these patients. An increase in circulating PANDER levels could be associated with the progression of T2D.

It has been shown that PANDER interacts with some unknown proteins on the liver membrane. In fact, the liver PANDER receptor has still not been identified. PANDER binds to the cell membrane of mouse liver cells and human HepG2 cells, inducing IR (85). In HepG2 cells, pretreatment with PANDER significantly inhibited the activation of insulin-stimulated proteins involved in the insulin signaling pathway (86). Thus, these results suggest that the liver is a novel target for islet-secreted PANDER. Transgenic mice with PDX-1-driven specific overexpression of PANDER in pancreatic β-cells developed fasting hyperglycemia, liver IR and increased steatosis (87). PANDER mRNA and protein expression were increased in the livers of HFD-fed mice and db/db mice. In contrast, hepatic PANDER silencing improved global IR and steatosis in db/db mice (88).

Overall, chronic hyperglycemia and IR induce a compensatory increase in insulin secretion. As PANDER is co-secreted with insulin, this may result in an increased production of PANDER in islets. Excess PANDER negatively affects GSIS. Moreover, PANDER binds liver cells and increases steatosis and hepatic glucose metabolism, generating a higher IR. Thus, PANDER has been proposed as a novel linker between IR and T2D suggesting that circulating PANDER could be a novel biomarker for NAFLD and T2D and could represent a novel strategy for the treatment of both diseases.

## EVs Also Contributes to Liver and Pancreatic Islet Crosstalk

During the last decade, plenty of studies have shown the contribution of EVs as mediators of organ crosstalk. EVs, that include microvesicles and exosomes, carry different bioactive cargo of proteins, lipids, metabolites, DNA and RNA (particularly micro RNAs (miRNAs) and small regulatory RNAs).

More recently, EVs have been shown to participate in metabolic diseases, such as NAFLD, IR and T2D (89). One important aspect of the role of EVs in the dialogue is that the miRNA cargo is able to modify the gene expression of tissues targets. This could mean that the different pathways organ crosstalk could be connected to each other and act in a synergistic way.

EVs release is frequently increased in liver diseases and often with a different cargo. This means that they are probably involved in the pathogenesis of liver alterations (90). In this regard, mouse, rat and human hepatocytes increased their EVs release when cultured in the presence of saturated fatty acids (90). Moreover, in NASH mouse models, it has been found a serum increase of EVs, together with a correlation with liver apoptosis and fibrosis (91). The majority of the cargo released by the EVs are related with liver physiology and pathophysiology. In fact, et al. using proteomic analysis from protein extracts of rat hepatocyte exosomes identified more than 251 different proteins, that could be assigned to diverse genome ontology categories (92).

Among the cargo released by liver EVs, several miRNAs can regulate different metabolic functions in several tissues like pancreatic β-cells. In this regards, several miRNA that are increased in the liver with NAFLD have been shown to regulate insulin release. For example, miRNA-375 (93), miRNA-9 (94) and miRNA-143 (95) decreased GSIS. Finally, miRNA-7218-5p, released from EVs derived from HFD induced obese mice promoted proliferation of the β-cell line MIN6 (96).

In the case of pancreatic islets, many studies have demonstrated the important role of exosomes in β-cell development, function and survival. In addition, modifications in miRNAs has been observed mice models of diabetes and in diabetic patients (97). As happens with hepatocytes, many of the exosomal miRNAs are transferred between β-cells forming a communications network (98). Nevertheless, some exosomal miRNAs derived from β-cells also exerts effects on hepatocytes. Xu et al. showed that miRNA-26a not only modulate insulin secretion and β-cell replication but also prevents obesity-induced metabolic liver alterations and IR (99). Finally, miRNA-29s has been shown to regulate liver insulin sensitivity and control glucose homeostasis (100).

The field of EVs is rapidly evolving and the role of their cargo, mainly miRNAs, in the regulation of glucose homeostasis through their participation in hepatocytes and β-cells crosstalk is becoming increasingly important.

## Discussion

In this review, we focused on analyzing the crosstalk between pancreatic β-cells and the liver and how this communication affects pancreatic β-cell physiology. In this sense, we found that pancreatic β-cells do not live isolated from their environment, and in addition to establishing relationships with other cell lines of the pancreatic islets, they also do so with the liver tissue, with which they share the important function of regulating metabolism. In summary, we found that proteins and factors released by the liver contribute to regulating the pathophysiology of pancreatic β-cells. Similarly, factors released by pancreatic β-cells act on the liver, regulating the metabolic functions of this organ.

**Figure 1:**
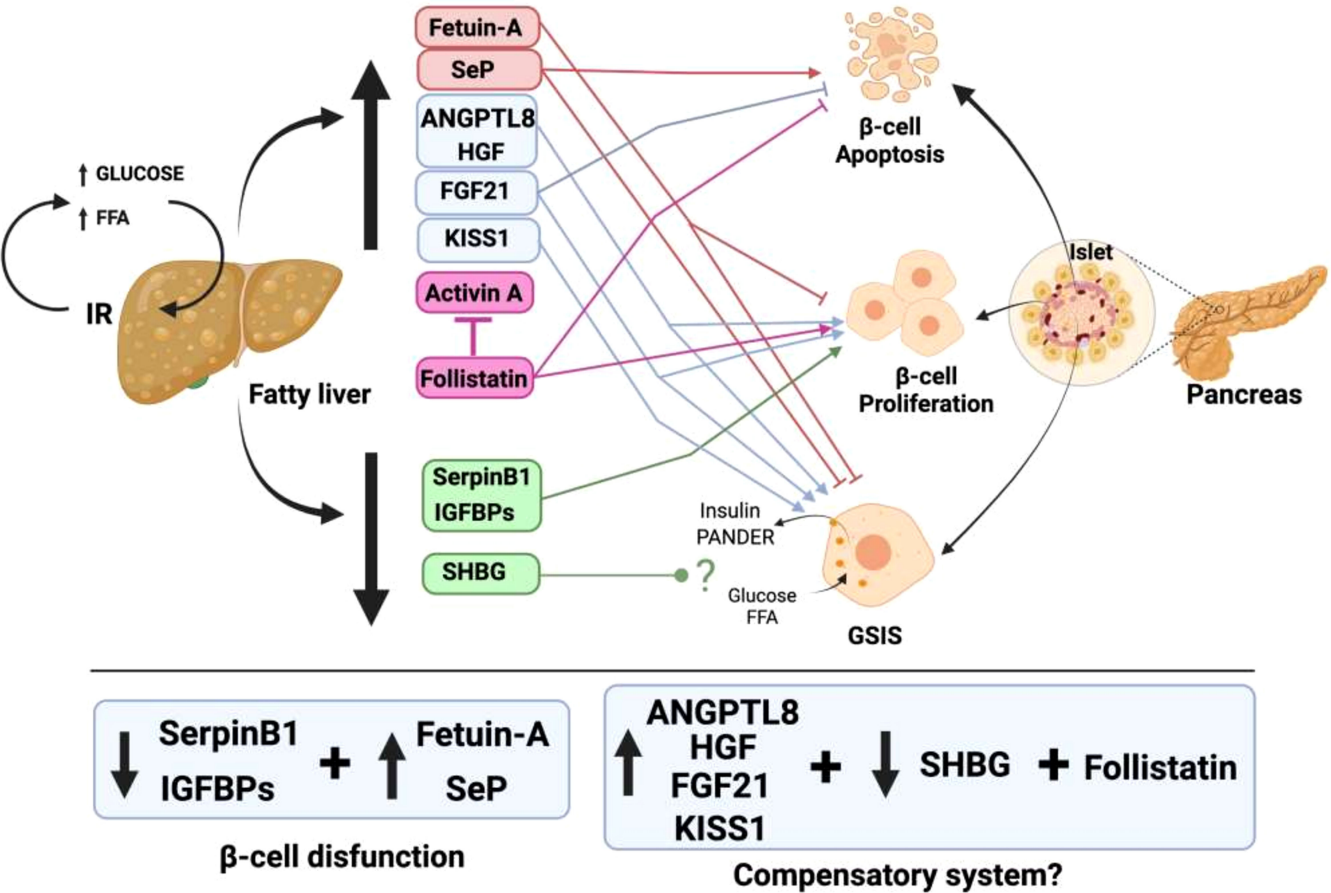
Liver factors involved in the talk of hepatic tissue with pancreatic β-cells in the presence of fatty liver. In conditions of nutrient excess, a fatty liver is produced, together with IR. This fatty liver generates an increased release of hepatokines that have deleterious effects on pancreatic β-cells (red box). In addition, there is an increase in other hepatokines that trigger compensatory mechanisms of increased β-cell proliferation and GSIS enhancement to try to fight IR (blue box). In contrast, there is a decrease in the release of other hepatokines that produce an increase in β-cell proliferation to compensate for the dysfunction that exists in them (green box).

**Figure 2:**
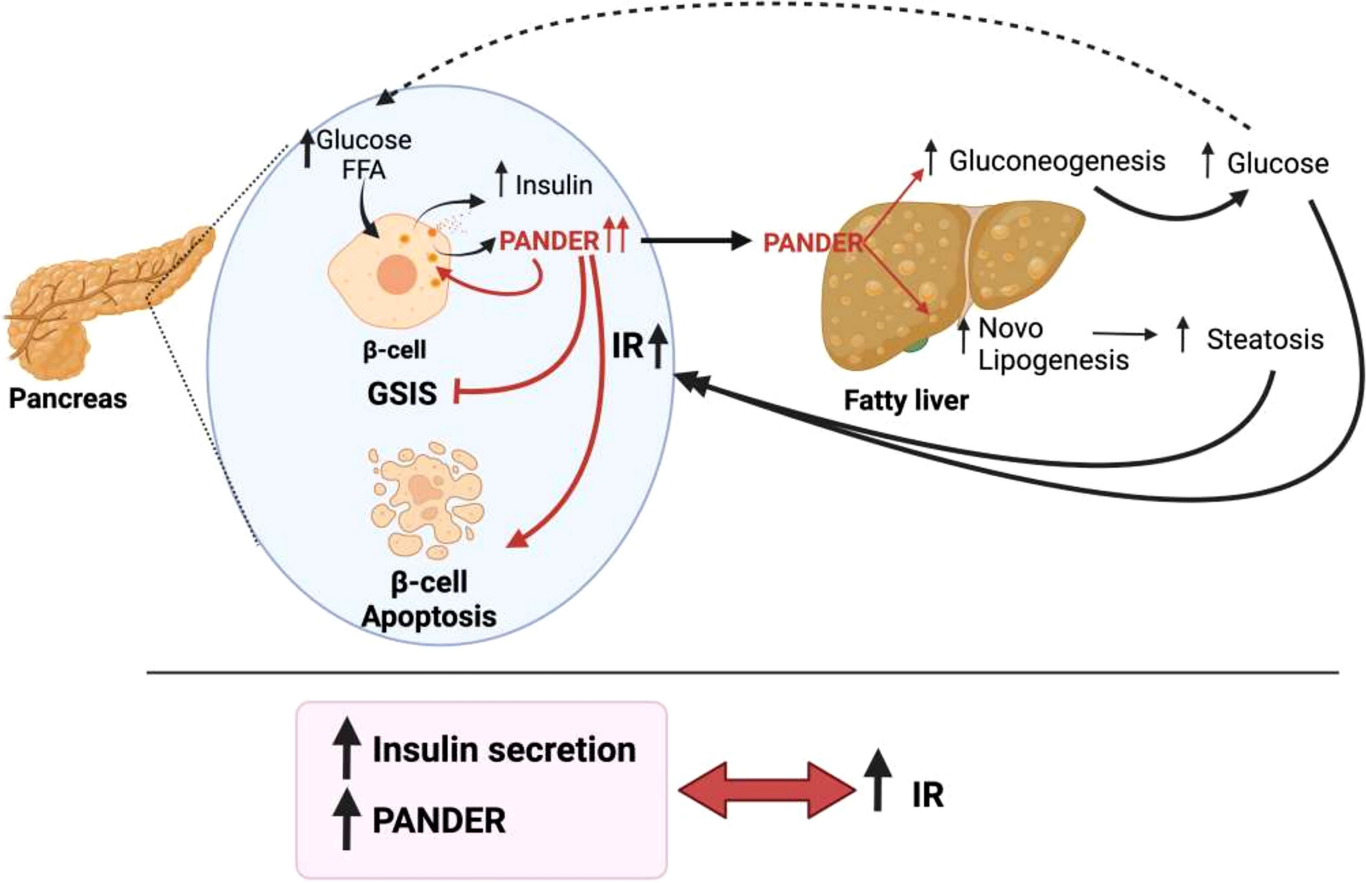
Pancreatic derived factor (PANDER) is the only word used by pancreatic β-cells to communicate with the liver. In the presence of nutrients, PANDER is co-secreted with insulin and acts to regulate insulin release. When IR is present, β-cells increase insulin and PANDER release. In this situation, the increase in PANDER induces β-cell apoptosis and a decrease in GSIS. In addition, PANDER travels to the liver, binds to its receptors on hepatocyte membranes and leads to increased gluconeogenesis and steatosis.

Notably, although there is fluid communication between the liver and pancreatic β-cells, the same does not occur in the opposite direction. For the liver, up to nine proteins and factors involved in crosstalk with pancreatic islets have been described (**Figure 1**). In contrast, only one protein released by β-cells that acts on the liver has been found thus far, and furthermore, the hepatic receptor to which this protein binds is not known (**Figure 2**). The explanation for this could be that pancreatic β-cells are the nerve center of communication between both tissues. However, it is also plausible that more signaling molecules originating in pancreatic β-cells have not yet been found.

**Figure 3:**
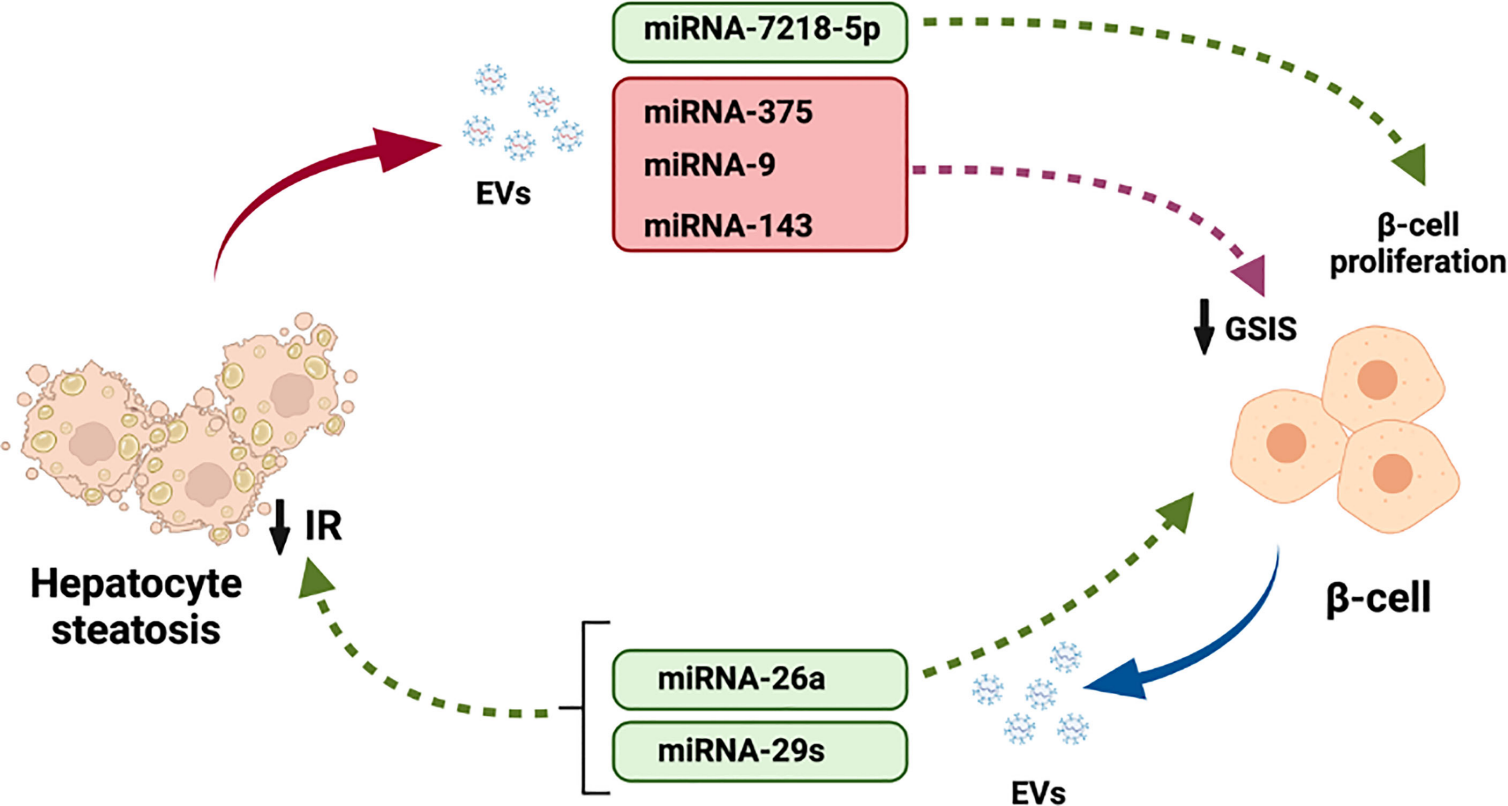
Participation of EVs cargo in the crosstalk between hepatocytes and pancreatic β-cells. Steatotic hepatocytes release EVs with miRNAs-375, 9 and 143 that reduced β-cell GSIS. Moreover, miRNA-7218-5p released by fatty liver promotes β-cell proliferation. In addition, miRNAs-26a and 29s released by β-cell EVs regulate liver IR.

In the last 5 years, EVs have strongly entered in the scene of hepatocyte- β cell crosstalk. The majority of the cargo released by EVs in the liver and pancreatic islets mostly serves to regulate the physiology and pathophysiology of both tissues. However, increasing evidence is accumulating for the involvement of molecules released by EVs, particularly miRNAs, in the dialogue between both tissues (**Figure 3**). This new data suggests that there exist potential for a bigger importance of EV-mediated crosstalk, since nearly all tissues in the body release EVs. Further research is needed to understand how EVs cargo will affect the complex crosstalk between hepatocytes and β-cells.

Another aspect that is important to discuss is that the proteins that participate in the dialog are released by both tissues in a different way in pathological situations, and even their role in the regulation of both tissues is different. Thus, it important to further study the crosstalk between liver and pancreatic β-cells under conditions of T2D, steatosis and steatohepatitis. However, the role that drugs used for T2D treatment might play in the interaction between the liver and pancreatic β-cells has not been explored thus far.

Finally, in the coming years, we should investigate the crosstalk between the two organs, since there are many aspects of this dialog that remain unresolved. It is certain that β-cells are not alone in the task of regulating glycemia and that the input they receive from the liver is fundamental. However, the liver most likely needs the help of β-cells to contribute to the regulation of glucose homeostasis. In this sense, it will be discovered that islets are interactive structures connected with other tissues through intense communication. Advances in this knowledge will most likely translate into new developments for the prevention and treatment of metabolic diseases. Finally, a better understanding of the bidirectional conversations between the liver and pancreatic β-cells may lead to new therapeutic avenues aimed at preventing or improving the treatment of T2D and NAFLD.

## Data Availability Statement

The original contributions presented in the study are included in the article/supplementary material. Further inquiries can be directed to the corresponding authors.

## Author Contributions

All authors participated directly in the manuscript. FM and GB wrote the original manuscript. LL-B, AL-S, DM, RG-D, JA, and MR-G reviewed and edited the manuscript and figures. GB performed the digital art. All authors contributed to the article and approved the submitted version.

## Funding

Funding from the following sources was used for this manuscript: AGL2017-86927-R and PID2020-116731RB-C21 from Ministerio de Ciencia e Innovación to FM and PC-0148-2016-0148 from Junta de Andalucía to FM and RG-D.

## Conflict of Interest

The authors declare that the research was conducted in the absence of any commercial or financial relationships that could be construed as a potential conflict of interest.

## Publisher’s Note

All claims expressed in this article are solely those of the authors and do not necessarily represent those of their affiliated organizations, or those of the publisher, the editors and the reviewers. Any product that may be evaluated in this article, or claim that may be made by its manufacturer, is not guaranteed or endorsed by the publisher.
